# A systematic review of movement and muscular activity biomarkers to discriminate non-specific chronic low back pain patients from an asymptomatic population

**DOI:** 10.1038/s41598-021-84034-x

**Published:** 2021-03-12

**Authors:** Florent Moissenet, Kevin Rose-Dulcina, Stéphane Armand, Stéphane Genevay

**Affiliations:** 1 Kinesiology Laboratory, University of Geneva and Geneva University Hospitals, Geneva, Switzerland; 2 Department of Rheumatology, Geneva University Hospitals, Geneva, Switzerland

**Keywords:** Diagnostic markers, Musculoskeletal system

## Abstract

The identification of relevant and valid biomarkers to distinguish patients with non-specific chronic low back pain (NSCLBP) from an asymptomatic population in terms of musculoskeletal factors could contribute to patient follow-up and to evaluate therapeutic strategies. Several parameters related to movement and/or muscular activity impairments have been proposed in the literature in that respect. In this article, we propose a systematic and comprehensive review of these parameters (i.e. potential biomarkers) and related measurement properties. This systematic review (PROSPERO registration number: CRD42020144877) was conducted in Medline, Embase, and Web of Knowledge databases until July 2019. In the included studies, all movements or muscular activity parameters having demonstrated at least a moderate level of construct validity were defined as biomarkers, and their measurement properties were assessed. In total, 92 studies were included. This allowed to identify 121 movement and 150 muscular activity biomarkers. An extensive measurement properties assessment was found in 31 movement and 14 muscular activity biomarkers. On the whole, these biomarkers support the primary biomechanical concepts proposed for low back pain. However, a consensus concerning a robust and standardised biomechanical approach to assess low back pain is needed.

## Introduction

Low back pain is the leading cause of disability worldwide since 1990 with an increase in years lived with disability of over 50% since then^1^. In 85–90% of the cases, the exact cause of pain cannot be ascertained with certainty and patients are thus classified as having non-specific low back pain^2^. Among these patients, 10% become chronic sufferers (non-specific chronic low back pain—NSCLBP) which represent a high socioeconomic burden^3^. In the absence of a clear and convincing diagnosis, therapeutic management remains difficult^4^. Various treatments exist but show small to moderate overall effects, potentially due to a lack of knowledge in the pathophysiology of NSCLBP and to the heterogeneity of the studied population^5^.

To date, NSCLBP is often described as a complex disorder where central and peripheral nociceptive processes are influenced by various factors such as social, psychological or musculoskeletal factors which interact with each other^3,6^. It has been proposed that social and psychological factors may play an important role in the persistence of the pain^7,8^. However, the role of musculoskeletal factors remains unclear. Regularly considered as pain consequences, these factors could be voluntary or involuntary compensations deployed to reduce pain, leading to long-term nociceptive consequences^9^.

Several musculoskeletal factors related to movement and/or muscular activity impairments have been highlighted in NSCLBP patients compared to asymptomatic participants^10^. Kinematic parameters such as range of motion, segment coordination or movement variability^11,12^, as well as electromyographic parameters such as maximal activity, timing activity or fatigability^13,14^, are regularly measured in NSCLBP population. However, due to methodological differences and/or the heterogeneity of the NSCLBP population, some studies reported contradictory findings^15^. On this basis, it seems difficult to develop efficient therapeutic programs. To be efficient, in addition to social and psychological factors, programs have to focus on musculoskeletal factors in NSCLBP patients that can be measured and evaluated using biomarkers, i.e. measurable parameters giving objective indications of patient state, which can be measured accurately and reproducibly^16^. In other words, the measurement properties^17^ of these biomarkers have to be known in terms of reliability, validity, interpretability and responsiveness. However, to the best of our knowledge, a comprehensive review of these biomarkers and related measurement properties is missing in the recent literature.

As a first step toward this wide objective, the purpose of this systematic review was 1) to identify in the literature the primary biomarkers related to movement or muscular activity and 2) to report their reliability, validity, interpretability levels, when available. In this sense, this manuscript attempts to establish a list of relevant biomarkers allowing to discriminate NSCLBP patients from an asymptomatic population from a musculoskeletal factors point of view and to report their respective level of validation.

## Results

### Study selection

**Figure 1:**
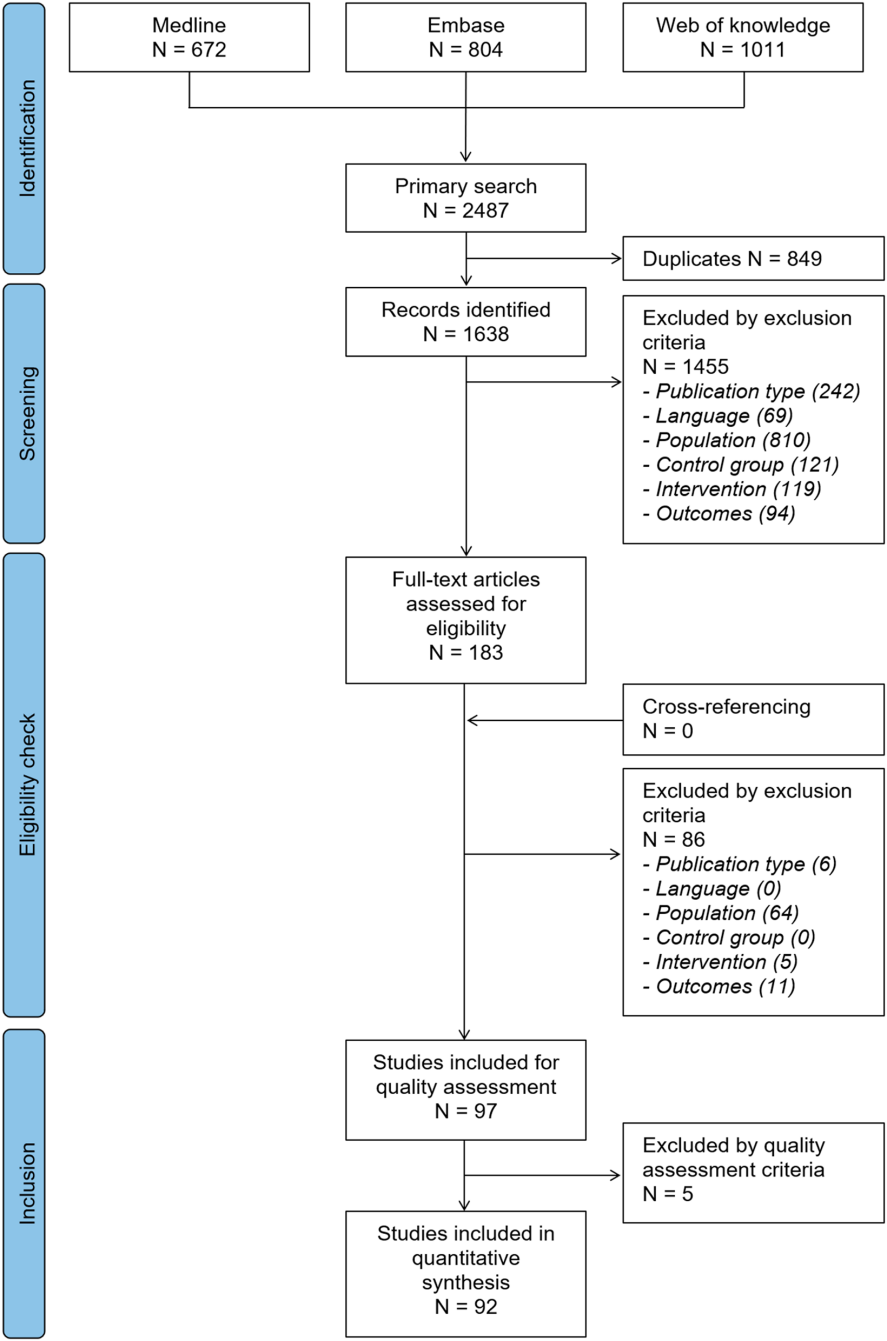
Flowchart of the search strategy conducted in this review (based on the PRISMA flowchart^47^).

The search strategy allowed to identify 672 records in Medline, 804 in Embase, and 1011 in Web of knowledge, yielding to 1638 records without any duplicate (Fig. 1).

According to the exclusion criteria, 97 studies were included for quality assessment (1455 records were cancelled after the screening of the abstract, and 85 after screening of the full-text article). Most of the excluded studies did not refer to adult patients (18–65-year-old) suffering from NSCLBP without a history of back surgery or pregnancy specifically (i.e. population criterion).

### Quality assessment

The overall score of each included study was calculated by the sum of rated criteria divided by the sum of applicable questions. The included studies were generally of good quality, with a mean score of 72 ± 12% (Supplementary Table 1). However, less than 50% of the studies provided sufficient information about their design or reported the reliability of the outcome, and less than 25% of the studies performed blinded analysis or justified the sample size. Five of the 97 studies^18–22^ included for quality assessment obtained a score lower than 9 (≤ 50%) and were thus not included in the data extraction process.

### Data extraction

**Figure 2:**
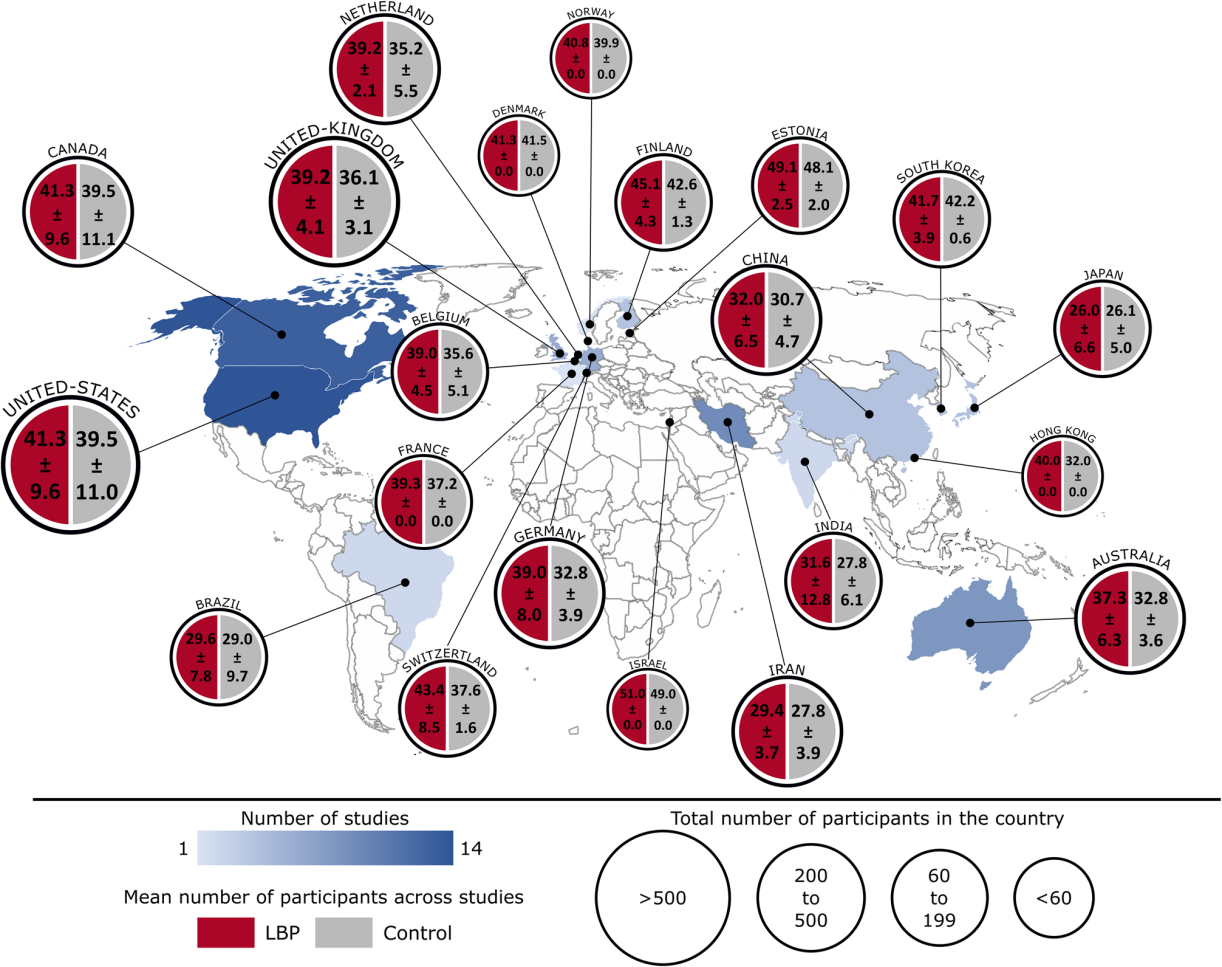
Labelled world map reporting the number of studies and the number of participants (NSCLBP and control) for each country of origin.

While not used in the data synthesis process, the full characteristics of the populations and measured parameters of each included study are available in Supplementary Table 2 to provide complete and transparent information, and a synthesis is all available in Fig. 2. The characteristics of each movement and muscular activity biomarker, used to conduct the data synthesis, are available in Supplementary Table 3.

### Data synthesis

As most of the biomarkers were only assessed by one study (see below), the risk to get heterogeneous populations from different studies to analyse one marker is low and was not considered in this analysis. Anyway, it can be observed that included studies come mainly from occidental Europe including UK (32%), North America (28%) and Asia (12%). The mean number of participants across studies was 29.0 ± 30.4 [min: 5, max: 218] for NSCLBP, and 23.1 ± 16.5 [min: 6, max: 130] for control. It can also be observed that a significant portion of the included studies did not report pain levels (20%), respectively functional disability score (35%) in NSCBPL patients. The full characteristics of the populations are available in Supplementary Table 2.

**Figure 3:**
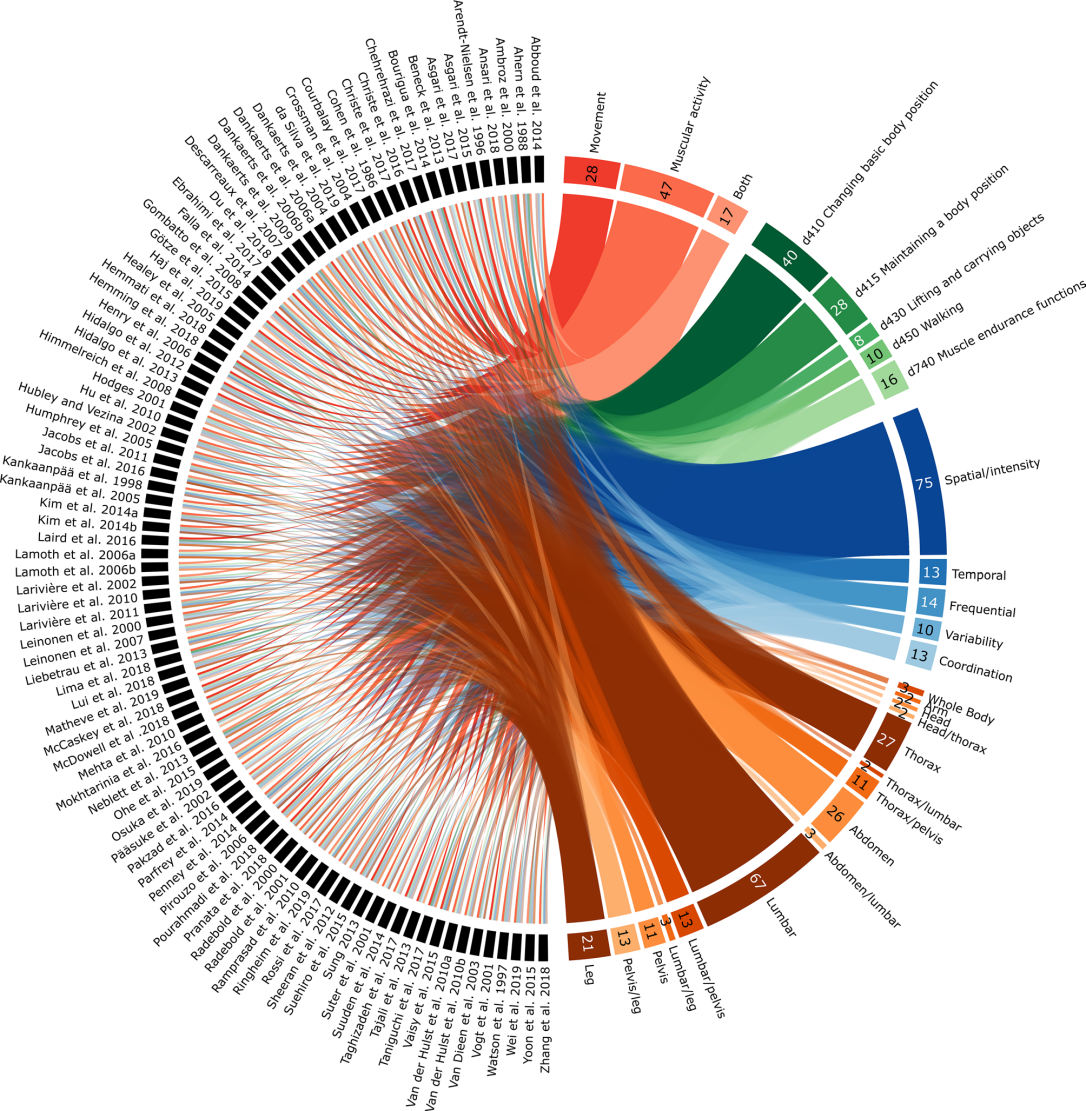
Circos plot^55^ linking the included studies to their selected parameter types, ICF 2nd level categories, variable categories and regions of interest. The number of studies linked to each item is also reported.

The Circos plot in Fig. 3 highlights the fact that muscular activity parameters were predominant in the included studies (70%: 51% only muscular activity parameters, 18% both movement and muscular activity parameters). The tasks related to these parameters were primarily ICF 2nd level category d410 “Changing basic body position” (43%), i.e. tasks with a movement excluding locomotion and weight lifting, and then d415 “Maintaining a body position” (30%), d740 “Muscle endurance functions” (17%), e.g. the Biering-Sørensen test, d450 “Walking” (11%) and d430 “Lifting and carrying objects” (9%). The variable related to these parameters were primarily spatial/intensity values (82%), and then frequential (15%), temporal (14%), coordination (14%) and variability values (11%). These variables were primarily targeted toward the lumbar region (43%), and then thorax (20%), pelvis (18%), legs (18%), abdomen (14%), head (2%), whole body (1%) and arms (1%) regions.

**Figure 4:**
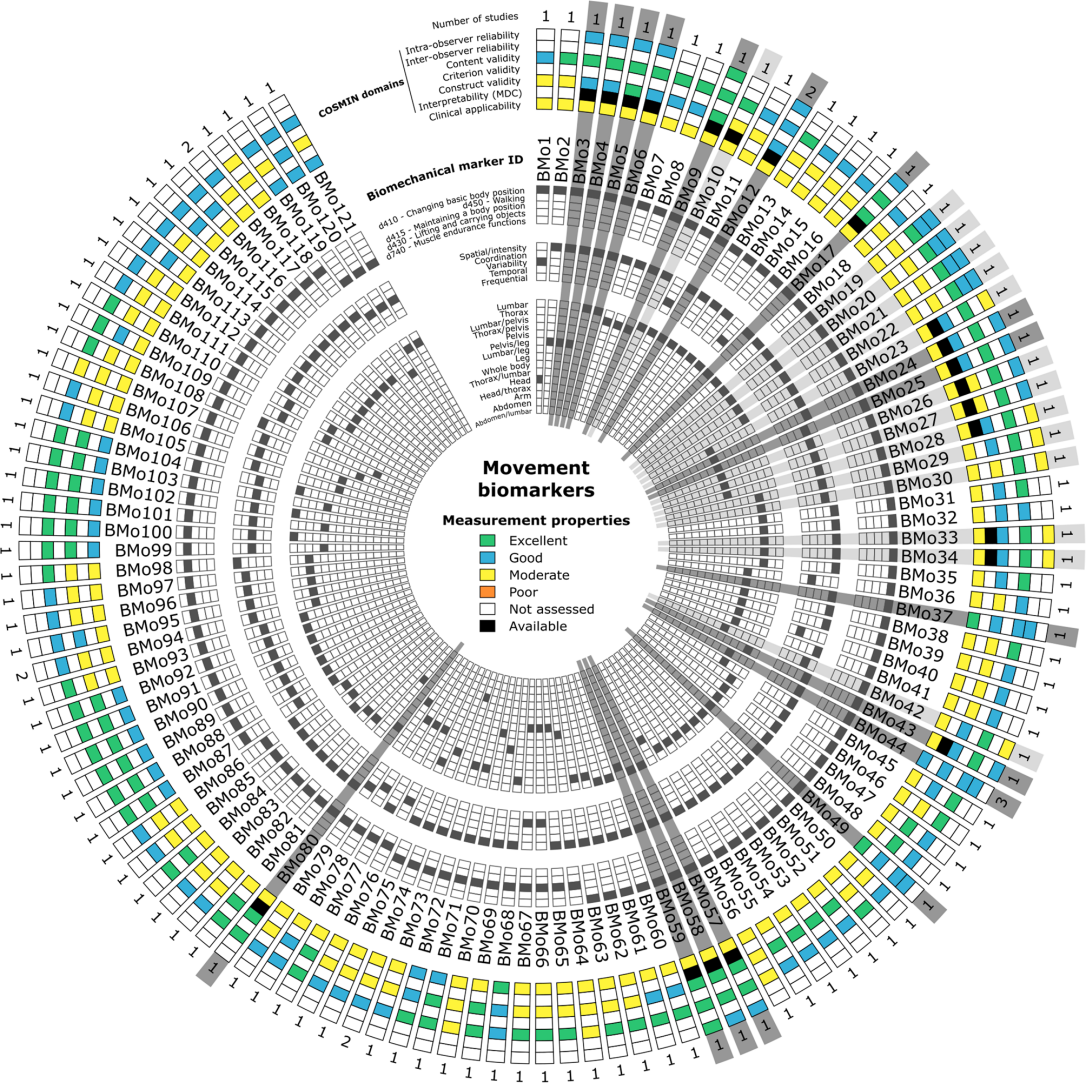
Circos plot^55^ synthesising the main characteristics and measurement properties of each movement biomarker. See Table 4 for measurement properties rating. Biomarkers are underlined in grey when at least 3 COSMIN items have been assessed, and dark grey when all these items reached at least a good level. Biomarker characteristics have been sorted by occurrence.

The Circos plot in Fig. 4 highlights first the fact that the measurement properties of the reported movement biomarkers were mainly assessed by only one study (96%), and then two studies (3%) or three studies (1%). Reliability was assessed in only 24% of these biomarkers (3% both intra- and inter-observer reliability, 18% intra-observer reliability only and 3% inter-observer reliability only). When considering altogether intra- and inter-observer reliability results, the reported level was generally good (73%), and then moderate (21%) and excellent (6%). Criterion validity was never assessed. Content validity was generally good (55%) or excellent (41%), but construct validity was mainly moderate (48%) and then excellent (27%) and good (25%). Interpretability (MDC) was assessed for only 21 biomarkers (17%). For a large majority of biomarkers, the clinical applicability, regarding the protocol used in the included studies, was moderate (83%). Biomarkers with at least three assessed COSMIN items have been underlined in grey in Fig. 4. Only 31 biomarkers were underlined (26% of all movement biomarkers) and reported in Table 1. It can be noticed that these biomarkers are mainly (97%) related to the ICF 2nd level category d410 “Changing basic body position” and mainly (77%) related to spatial/intensity variables and lumbar region (70%: 35% lumbar, 19% lumbar/leg, 16% lumbar/pelvis). Seventeen of these markers (i.e. 14% of all movement biomarkers) reached at least a good level in the assessed COSMIN items (underlined in dark grey in Fig. 4).

**Table 1:** List of movement biomarkers having at least 3 COSMIN items assessed in the included studies. Biomarkers for which all these items reached at least a good level (see Table 4 for measurement properties rating) are in bold.

ID	Variable name	Task	Reliability		Validity			Interpr	Clinical appl	Study
			R1	R2	V1	V2	V3	MDC		
**BMo3**	**Hip sagittal angle (rom)**	**Sit to stand**	**+/++**	**NA**	**+++**	**NA**	**++**	**●**	**+**	**Pourahmadi et al., 2018**^57^
**BMo4**	**Hip sagittal angle (rom)**	**Stand to sit**	**++/++**	**NA**	**+++**	**NA**	**++**	**●**	**+**	**Pourahmadi et al., 2018**^57^
**BMo5**	**Lower lumbar sagittal angle (max)**	**Trunk sagittal bending**	**+++/++**	**NA**	**+++**	**NA**	**+++**	**●**	**+**	**Hidalgo et al., 2012**^25^
**BMo6**	**Lower lumbar sagittal angular velocity (max)**	**Trunk sagittal bending**	**+++/++**	**NA**	**+++**	**NA**	**++**	**●**	**+**	**Hidalgo et al., 2012**^25^
**BMo9**	**Lower thorax sagittal angle (max)**	**Trunk sagittal bending**	**++/+++**	**NA**	**+++**	**NA**	**+++**	**●**	**+**	**Hidalgo et al., 2012**^25^
BMo10	Lumbar contribution to thorax angle (rom)	Trunk sagittal bending	NA	NA	+++	NA	+	●	+	Laird et al., 2016^42^
**BMo12**	**Lumbar sagittal angle (max)**	**Trunk sagittal bending**	**+++/++**	**NA**	**++**	**NA**	**++**	**●**	**+**	**Hidalgo et al., 2012**^25^, **2013**^58^
**BMo17**	**Lumbar sagittal angular velocity (max)**	**Trunk sagittal bending**	**+++/++**	**NA**	**+++**	**NA**	**+++**	**●**	**+**	**Hidalgo et al., 2012**^25^
BMo19	Lumbar transversal angle (max)	Trunk rotation	++/++	++/++	+++	NA	+	NA	+	Haj et al., 2019^59^
BMo20	Lumbar transversal angular acceleration (max)	Trunk rotation	++/++	++/++	+++	NA	+	NA	+	Haj et al., 2019^59^
BMo21	Lumbar transversal angular velocity (max)	Trunk rotation	++/++	++/++	+++	NA	+	NA	+	Haj et al., 2019^59^
BMo22	Lumbar transversal angular velocity (mean)	Trunk rotation	++/++	++/++	+++	NA	+	NA	+	Haj et al., 2019^59^
BMo23	Lumbar/hip ratio of sagittal angle (rom)	Sit to stand	+/+	NA	+++	NA	++	●	+	Pourahmadi et al., 2018^57^
**BMo24**	**Lumbar/hip ratio of sagittal angle (rom)**	**Stand to sit**	**+/++**	**NA**	**+++**	**NA**	**++**	**●**	**+**	**Pourahmadi et al., 2018**^57^
**BMo25**	**Lumbar/hip relative phase difference (max)**	**Sit to stand**	**++/++**	**NA**	**+++**	**NA**	**++**	**●**	**+**	**Pourahmadi et al., 2018**^57^
BMo26	Lumbar/hip relative phase difference (mean)	Sit to stand	+/++	NA	+++	NA	+	●	+	Pourahmadi et al., 2018^57^
BMo27	Lumbar/hip relative phase difference (mean)	Stand to sit	+/++	NA	+++	NA	+	●	+	Pourahmadi et al., 2018^57^
BMo28	Lumbar/hip relative phase difference (min)	Sit to stand	+/+	NA	+++	NA	++	●	+	Pourahmadi et al., 2018^57^
BMo29	Lumbar/pelvis absolute relative phase (mean)	Trunk sagittal bending	+/+	NA	+++	NA	++	NA	+	Mokhtarinia et al., 2016^60^
BMo30	Lumbar/pelvis deviation phase (mean)	Trunk sagittal bending	+/+	NA	+++	NA	+	NA	+	Mokhtarinia et al., 2016^60^
BMo33	Lumbopelvic sagittal angle (rom)	Sit to stand	++/+	NA	+++	NA	++	●	+	Pourahmadi et al., 2018^57^
BMo34	Lumbopelvic sagittal angle (rom)	Stand to sit	+/+	NA	+++	NA	++	●	+	Pourahmadi et al., 2018^57^
**BMo37**	**Pelvis sagittal angle (max)**	**Trunk sagittal bending**	**NA**	**++/++**	**++**	**NA**	**++**	**NA**	**+++**	**Neblett et al., 2013**^37^
BMo42	Pelvis/thigh deviation phase (mean)	Trunk sagittal bending	+/+	NA	+++	NA	++	NA	+	Mokhtarinia et al., 2016^60^
**BMo43**	**Scapular belt transversal angle (max)**	**Trunk rotation**	**++/++**	**NA**	**+++**	**NA**	**++**	**●**	**+**	**Hidalgo et al., 2012**^25^
**BMo44**	**Thoracopelvic sagittal angle (max)**	**Trunk sagittal bending**	**NA**	**++/++**	**++**	**NA**	**++**	**NA**	**++**	**Ahern et al., 1988**^61^; **Larivière et al., 2011**^62^; **Neblett et al., 2013**^37^
**BMo49**	**Thorax sagittal angle (max)**	**Trunk sagittal bending**	**NA**	**++/++**	**++**	**NA**	**++**	**NA**	**+++**	**Neblett et al., 2013**^37^
**BMo57**	**Upper lumbar sagittal angle (max)**	**Trunk sagittal bending**	**+++/++**	**NA**	**+++**	**NA**	**+++**	**●**	**+**	**Hidalgo et al., 2012**^25^
**BMo58**	**Upper lumbar sagittal angular velocity (max)**	**Trunk sagittal bending**	**+++/++**	**NA**	**+++**	**NA**	**+++**	**●**	**+**	**Hidalgo et al., 2012**^25^
**BMo59**	**Upper thorax sagittal angle (max)**	**Trunk sagittal bending**	**++/+++**	**NA**	**+++**	**NA**	**+++**	**●**	**+**	**Hidalgo et al., 2012**^25^
**BMo80**	**Lumbopelvic sagittal angle (max)**	**Trunk sagittal bending**	**+++/NA**	**NA**	**+++**	**NA**	**+++**	**●**	**+**	**Matheve et al., 2019**^63^

R1: Intra-observer reliability (controls/patients); R2: Inter-observer reliability (controls/patients); V1: Content validity; V2: Criterion validity; V3: Construct validity, rom: range of motion, max: maximum.

min: minimum, MDC: minimum detectable change, interp.: interpretability, appl.: applicability, in bold: biomarkers for which all items reached at least a good level (see Table 1 for measurement properties rating).

+++: Excellent; ++: Good; +: Moderate; ●: available; NA: not available (see Table 4 for measurement properties rating).

**Figure 5:**
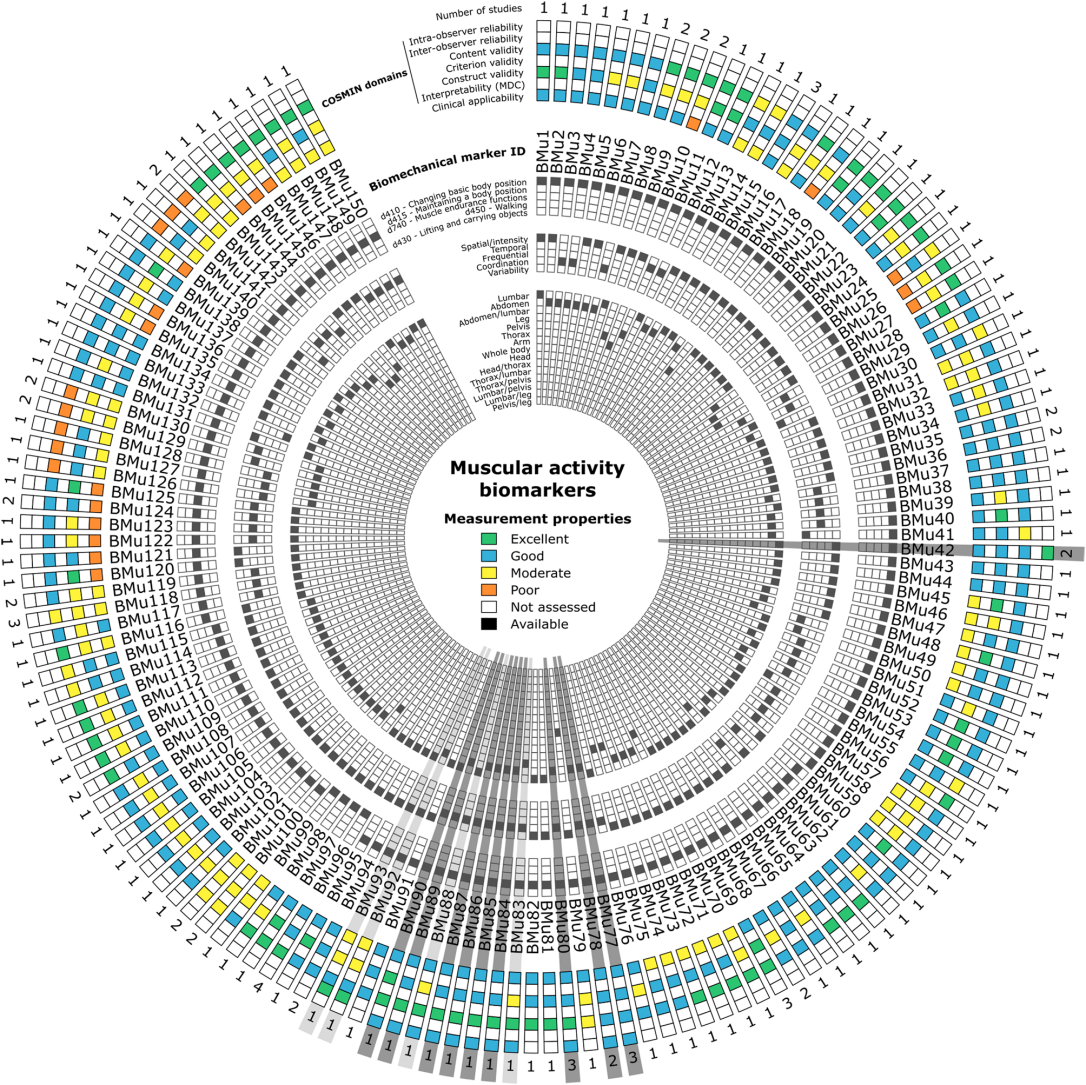
Circos plot^55^ synthesising the main characteristics and measurement properties of each muscular activity biomarker. See Table 4 for measurement properties rating. Biomarkers are underlined in grey when at least 3 COSMIN items have been assessed, and dark grey when all these items reached at least a good level. Biomarker characteristics have been sorted by occurrence.

The Circos plot in Fig. 5 highlights first the fact that the measurement properties of the reported muscular activity biomechanical markers were mainly assessed by one study (85%), and then two studies (11%), three studies (3%) or four studies (1%). Reliability was assessed in only 14% of these markers (0% both intra- and inter-observer reliability, 8% intra-observer reliability only and 1% inter-observer reliability only). When considering altogether intra- and inter-observer reliability results, the reported level was good (79%) or excellent (21%). Criterion validity was never assessed. Content validity was generally good (53%), and then excellent (34%), moderate (8%) and poor (5%). Construct validity was mainly moderate (47%) and then good (39%) and excellent (14%). Interpretability (MDC) was never assessed. Clinical applicability, regarding the protocol used in the included studies, was generally good (62%), and then moderate (27%) and poor (11%). Markers with at least three assessed COSMIN items have been underlined in grey in Fig. 5 and reported in Table 2. Only 14 markers were identified (9% of all identified muscular activity biomechanical markers). It can be noticed that these markers are mainly (93%) related to the ICF 2nd level category d415 “Maintaining a body position” and mainly (79%) related to temporal variables and abdomen/lumbar region (90%: 45% lumbar, 38% abdomen, 7% abdomen/lumbar). Ten of these markers (i.e. 7% of all identified muscular activity biomechanical markers) reached at least a good level in the assessed COSMIN items (underlined in dark grey in Fig. 5).

**Table 2:** List of muscular activity biomarkers having at least 3 COSMIN items assessed in the included studies. Biomarkers for which all these items reached at least a good level (see Table 4 for measurement properties rating) are in bold.

ID	Variable name	Task	Reliability		Validity			Interpr	Clinical appl	Study
			R1	R2	V1	V2	V3	MDC		
**BMu42**	**Erector spinae (longissimus) EMG signal (max) flexion/maximal flexion ratio**	**Trunk sagittal bending**	**NA/+++**	**NA**	**++**	**NA**	**++**	**NA**	**++**	**Neblett et al., 2013**^37^; **Watson et al., 1997**^38^
**BMu77**	**Rectus abdominis activation onset latency**	**Two-legged standing**	**++/++**	**NA**	**++**	**NA**	**++**	**NA**	**++**	**Jacobs et al., 2016**^64^; **Liebetrau et al., 2013**^33^; **Mehta et al., 2010**^32^
**BMu78**	**External oblique activation onset latency**	**Two-legged standing**	**++/++**	**NA**	**++**	**NA**	**++**	**NA**	**++**	**Jacobs et al., 2016**^64^; **Mehta et al., 2010**^32^
**BMu80**	**Internal oblique activation onset latency**	**Two-legged standing**	**++/++**	**NA**	**+++**	**NA**	**++**	**NA**	**++**	**Liebetrau et al., 2013**^33^; **Mehta et al., 2010**^32^; **Osuka et al., 2019**^65^
BMu83	Internal oblique/multifidus cocontraction duration	Two-legged standing	++/++	NA	+++	NA	+	NA	++	Mehta et al., 2010^32^
**BMu84**	**Internal oblique activation burst duration**	**Two-legged standing**	**++/++**	**NA**	**+++**	**NA**	**++**	**NA**	**++**	**Mehta et al., 2010**^32^
**BMu85**	**Transversus abdominis activation burst duration**	**Two-legged standing**	**++/++**	**NA**	**+++**	**NA**	**++**	**NA**	**++**	**Mehta et al., 2010**^32^
**BMu86**	**External oblique activation burst duration**	**Two-legged standing**	**++/++**	**NA**	**+++**	**NA**	**++**	**NA**	**++**	**Mehta et al., 2010**^32^
**BMu87**	**Rectus abdominis activation burst duration**	**Two-legged standing**	**++/++**	**NA**	**+++**	**NA**	**++**	**NA**	**++**	**Mehta et al., 2010**^32^
BMu88	Multifidus activation burst duration	Two-legged standing	++/++	NA	+++	NA	+	NA	++	Mehta et al., 2010^32^
**BMu89**	**Transversus abdominis activation onset latency**	**Two-legged standing**	**++/++**	**NA**	**+++**	**NA**	**++**	**NA**	**++**	**Mehta et al., 2010**^32^
**BMu90**	**Multifidus activation onset latency**	**Two-legged standing**	**++/++**	**NA**	**+++**	**NA**	**+++**	**NA**	**++**	**Mehta et al., 2010**^32^
BMu92	Rectus abdominis preprogrammed reaction amplitude	Two-legged standing	NA	+++/+++	++	NA	+	NA	+	Ramprasad et al., 2010^66^
BMu93	Erector spinae (longissimus) preprogrammed reaction amplitude	Two-legged standing	NA	+++/+++	++	NA	+	NA	+	Ramprasad et al., 2010^66^

R1: Intra-observer reliability (controls/patients); R2: Inter-observer reliability (controls/patients); V1: Content validity; V2: Criterion validity; V3: Construct validity.

max: maximum, MDC: minimum detectable change, interp.: interpretability, appl.: applicability, in bold: biomarkers for which all items reached at least a good level (see Table 1 for measurement properties rating).

+++: Excellent; ++: Good; +: Moderate; ●: available; NA: not available (see Table 4 for measurement properties rating).

## Discussion

In a recent special issue on low back pain^12^, the need to defined further quantitative biomarkers for low-back pain, including biomechanical parameters, has been pointed out. In line with this suggestion, this systematic review aimed to identify movement and muscular activity biomarkers proposed in the literature to discriminate NSCLBP patients from an asymptomatic population. The main findings are:

- Reported biomarkers were related to various tasks mostly measuring spatial or intensity values targeted to the lower back;
- Biomarkers were mostly (90%) reported in only one study for each of them, and only 8% of them were assessed in terms of reliability, validity and interpretability;
- Identified movement biomarkers measurement properties: inter-intra reliability, when assessed (24%), is good, construct validity is at least moderate, interpretability is rarely reported (17%), and clinical applicability is moderate;
- Identified muscular activity biomarkers measurement properties: inter-intra reliability, when assessed (14%), is good to excellent, construct validity is at least moderate, no study found on interpretability, and clinical applicability is generally good;
- Despite all this, we were able to identify 31 movement biomarkers and 14 muscular activity biomarkers for which an extensive measurement properties assessment is already available.

A first observation is the heterogeneous nature of the included studies, exploring a large variety of measures on almost all body regions and during various tasks. This heterogeneity illustrates the fact that there is still no consensus to define a clear protocol exploring the role of musculoskeletal factors in NSCLBP. This observation is in line with the diversity of biomechanical concepts in the literature^12^. A direct consequence is the split of the data reported in the literature, reducing chances to identify and validate one or several relevant biomarkers. Regarding the type of parameters, both movement and muscular activity parameters may lead to relevant information about NSCLBP, whatever the category of measured variables. Movements parameters have been mainly oriented, directly or indirectly, towards the intervertebral kinematics and can so be associated to the first biomechanical model “Intervertebral Mechanical Dysfunction in Nonspecific LBP” described by Cholewicki et al.^12^. Muscular activity parameters may also reflect the motor adaptation to the muscle disuse observed during the chronic phase and related to atrophy, fibrosis and fatty infiltration^14^. These parameters can be associated with the second “The Kinesiopathologic Model” and third biomechanical model “Anatomy, Biomechanics, and Pathology of the Sacroiliac Joints” described by Cholewicki et al.^12^. Various tasks have also been used in the included studies. As already reported by van Dijk et al.^23^, the ICF 2nd level categories d410 “Changing basic body position”, d430 “Lifting and carrying objects” and d450 “Walking” often illustrate the proposed tasks, but also d415 “Maintaining a body position” and d740 “Muscle endurance functions”. Concerning the region of interest, the thoraco-lombo-pelvis region is unsurprisingly preferred, while other body regions have also been studies and may reflect posture adaptations due to spine instability in case on NSCLBP^24^. As a consequence, it appears that the included studies rarely use the same parameter of interest, i.e. a variable measured in the same experimental conditions and during a similar task. Only 10% of the movement and muscular activity biomarkers highlighted in this systematic review were reported by more than one included study, and only 3% by more than two included studies. However, to be recognised and applied, a biomarker has to be evaluated so as to demonstrate its accuracy and reproducibility. Furthermore, this complex and time-consuming process needs to be reevaluated to ensure the relevant aspect of a biomarker^16^. Following the COSMIN recommendations^17^, four domains have to be explored to fully assessed the measurement properties of any measurement instrument, i.e. reliability, validity, responsiveness and interpretability. But, only 8% of the biomarkers reported in this systematic review were assessed in terms of reliability, validity and interpretability (responsiveness was out of the scope of this review). This result supports the fact that the full assessment of the measurement properties of a biomarker may be challenging to manage by a unique study. Reliability alone is a time-consuming process requiring several measurement sessions per participant. Consequently, only the construct validity (i.e. a statistically significant difference between the value of the biomarker for a NSCLBP and a control group) was assessed for 83% of the highlighted biomarkers. Regarding the results of this systematic review, we believe that it would be much more efficient to complete the measurement properties assessment of biomarkers already proposed by other studies. Without such a global effort, it may be extremely difficult to identify relevant biomarkers related to musculoskeletal factors in NSCLBP.

The data synthesis highlighted 121 movement biomarkers having demonstrated a discriminative ability (i.e. construct validity). This result supports the fact that the biomechanical aspect of NSCLBP has been widely studied in the literature. Indeed, movements and postures may have a direct or indirect impact on NSLCBP^12^ and many studies have thus explored mechanical factors to compare a NSCLBP and a control group. However, an extensive measurement properties assessment has only been performed for 31 of these biomarkers. For these biomarkers, a consistent level of reliability and validity has already been demonstrated in the included studies. For all others, additional studies are needed to further explore their relevance as biomarkers able to discriminate NSCLBP patients from an asymptomatic population. A first group of movement biomarkers, for which an extensive measurement properties assessment has been performed, focused on spine kinematics during trunk sagittal bending or rotation. This group of biomarkers relates to the first biomechanical model “Intervertebral Mechanical Dysfunction in Nonspecific LBP” described by Cholewicki et al.^12^. While the direct intervertebral motion measurement requires invasive or ionising approaches (i.e. intracortical bone pins and fluoroscopy, respectively), these biomarkers measure an indirect intervertebral motion, e.g. thorax or lumbar absolute or relative kinematics. The level of thoracolumbar spine segmentation depends on the studies, varying from two (thorax, lumbar) to four (upper and lower thorax and lumbar). A higher level of segmentation allows for a more precise spine mobility assessment^25^, but results are prone to soft tissue artefact issue, especially during trunk rotation^26^. Moreover, this mechanical consideration must be interpreted cautiously since any alteration of the thorax or lumbar angles may result from asymptomatic structural degenerations (e.g. disc degeneration, ligament tightening) appearing with aging, or from psychological factors (e.g. kinesiophobia)^12^. A second group of movement biomarkers corresponds to an altered lumbar-hip coordination^27^. They correspond to measurements of hip sagittal angle or lumbar/hip phase shifting during sit-to-stand, stand-to-sit and trunk sagittal bending tasks (i.e. balance challenging tasks). This group can be related to another mechanical consideration in NSCLBP dealing with spine stability^24^. In particular, a decreased lumbar-pelvis coordination has been reported by several studies^28^. Through a trunk extensor muscle dysfunction is NSCLBP patients, this altered coordination induces co-contractions that can restrain lumbar spine motion^28^. Again, this mechanical consideration must be interpreted cautiously since the reduced range of motion observed in pelvis and hips during these tasks may also be explained by kinesiophobia and/or by a preventive mechanism against pain^28,29^, or a consequence of muscle disuse, leading to muscle atrophy^14^.

The data synthesis highlighted 150 muscular activity biomarkers having demonstrated a discriminative ability. This result supports the fact that altered muscle function in NSCLBP has been widely studied in the literature. Indeed, several models such as the pain-spasm-pain model^30,31^ have been proposed, arguing for an impact of the muscle function on pain. Changes in the back-muscle structure (e.g. atrophy, fibrosis and fatty infiltration) have also been widely reported^14^. However, a measurement properties assessment has only been performed for 14 of these biomarkers. Analysis of the studies included in this review led to the conclusion that a consistent level of reliability and validity of these biomarkers has already been established. For all others, further analysis will be necessary to establish or not if they can be recognised or not as relevant biomarkers to discriminate NSCLBP patients from an asymptomatic population. A first group of muscular activity biomarkers for which an advanced measurement properties assessment has been performed corresponds to muscle activity adaptations under perturbations. They correspond to temporal variables (activation onset latency, activation burst duration, co-contraction duration) or spatial/intensity variables (EMG signal amplitude) during a postural task (two-legged standing) under expected^32^ or unexpected^33,34^ perturbations. Indeed, several authors have reported an impairment of the trunk postural control in NSCLBP patients^33^. Two strategies have been described in the literature to manage the trunk postural control, i.e. the anticipatory postural adjustment and the compensatory postural adjustment^35^, and related dysfunctions have been reported by several studies^32^. In this sense, this group of biomarkers could also be related to spine stability issues^24^. A particular emphasis, however, should be considered about temporal variables (e.g. activation onset latency, activation burst duration). Indeed, Mehta et al.^35^ highlighted that these variables might be sensitive to the high individual and between subject variation observed in EMG signal patterns. Furthermore, computational algorithms used for activation onset detection may vary in term of accuracy^36^. A second group of muscular activity biomarkers corresponds to the flexion/maximal flexion ratio of the erector spinae (longissimus) EMG signal during trunk sagittal bending^37,38^. This biomarker is related to the flexion relaxation phenomenon (FRP) well reported in the literature^39^. This phenomenon has been defined as a reduced myoelectric activity of the lumbar erector spinae longissimus (and multifidus) during full trunk sagittal bending. However, the EMG signal processing needed to compute the ratios related to this phenomenon is known to be very sensitive to the trunk sagittal bending velocity^40^ and the FRP temporal limits, defined by visual identification or automated methods^39^. Further reliability studies, including NSCLBP patients and asymptomatic subjects, should thus be considered to clarify the robustness of such a biomarker.

While EMG measurements have been considered in this systematic review as a practice already well established in clinical practice, most of the movement biomarkers were measured using complex and costly devices such as optoelectronic cameras with reflective markers. Thus, a majority of the highlighted biomarkers did not reach a good level of clinical applicability. However, except for the EMG exploration of deep muscles that requires intramuscular measures, most of the biomarkers that have been recorded using advance measurement instruments, in a dedicated laboratory, could be recorded using simpler, transportable, and less costly devices. For example, inertial measurement units (IMUs) are more and more extensively used in the field of biomechanics to spatiotemporal and kinematic parameters^41^. Such devices may open new avenues for the diagnosis, treatment and follow up of NSCLBP patients in clinical routine. Unfortunately, the measurement properties of any biomarkers should be re-evaluated for each measurement instrument, since all sensors have their own reliability and validity. Some authors have already focused their developments and analysis towards sensors with a high level of clinical applicability^42^. However, further studies will be required to transfer the biomarkers highlighted in this systematic review to clinical routine.

Our results must be interpreted carefully since this work has several limitations. First, even if the quality assessment was performed using a checklist adapted and validated for quantitative studies^43^, it was not necessarily adapted to evaluate the quality of laboratory studies involving various type of measurement tools (e.g. EMG, optoelectronic cameras). However, this checklist has already been used in the context of low back pain^44^ and was considered to be the most adapted to the needs of this systematic review. Second, the large number of studies included in this systematic review limited the possibilities to explore all methodological details. In order to highlight biomarkers and guide future research, several extracted data have been voluntarily omitted or simplified during the data synthesis process (e.g. task perturbations, EMG electrode placement). However, all extracted data are provided as Supplementary material to allow future data analysis. Third, the responsiveness of the highlighted biomarkers, i.e. their ability to detect change over time^17^, was not analysed. Since the aim of this systematic review was to identify movement and muscular activity biomarkers that can be used to discriminate NSCLBP patients from an asymptomatic population, this COSMIN domain was out of the present scope. However, as responsiveness may be one of the most important endpoint in clinical practice, it will be crucial to verify the sensitivity to change of the highlighted biomarkers and perform additional responsiveness studies when needed.

Several authors have pointed out the importance of having reliable biomarkers sustaining the biomechanical concepts proposed for low back pain^45^. Integrating these biomarkers into studies along with well recognised social and psychological factors has the potential to add to our understanding of this complex disease and to open the scientific community to new therapeutical approaches. This systematic review highlights that, even if several relevant biomarkers related to movement and muscular activity have been proposed and their measurement properties partially assessed, there is currently a lack of consensus concerning a robust and standardised biomechanical approach to assess low back pain. Prior to such a consensus, it is however crucial to increase the current knowledge on the biomarkers highlighted here (and on any other possible biomarker) to ascertain that all COSMIN domains (i.e. reliability, validity, responsiveness and interpretability) have been well explored. For that, future studies should seriously consider reproducing existing protocols and measure parameters in the same conditions than in the original articles, but also in different countries, cultures and pain/disability levels on low back pain populations. The use of sensors known for a high clinical applicability should also be further deployed to ease the appropriation of the related measurements in clinical routine. Finally, every study should report pain level and disability score of the included patients to better characterise the assessed populations and possibly allow the identification of different sub-groups within this heterogeneous population.

This systematic review highlighted biomarkers related to movement and muscular activity allowing to discriminate NSCLBP patients from an asymptomatic population from a musculoskeletal factors point of view. While numerous parameters were assessed in the literature, with a large heterogeneity and mainly one study for one measurement, a comprehensive assessment of the measurement properties of 31 movement biomarkers and 14 muscular activity biomarkers was identified in the included studies. On the whole, these biomarkers support the primary biomechanical concepts proposed for low back pain. However, a consensus concerning a robust and standardised biomechanical approach to assess low back pain is currently missing but desperetly needed in order to improve our knowledge on this condition and extend our therapeutic capabilites.

## Methods

### Study design

This study is a systematic review based on the following research question established using the PICO approach^46^: *“In adults suffering non-specific chronic low back pain, what are the biomarkers that allow to discriminate them from an asymptomatic population in terms of movement or muscular activity?”*.

### Protocol and registration

This systematic review was registered through PROSPERO (registration number: CRD42020144877) before being undertaken. It has been written following the Preferred Reporting Items for Systematic review and Meta-Analysis (PRISMA) Statement^47^. The assessment of each reported outcome measurement instrument was inspired by the COSMIN checklist^17^.

### Information sources and search strategy

An electronic search was performed in Medline, Embase, and Web of Knowledge databases from inception to July 2019 without any time limit. The logical (nested) expressions for the search were: (’low* back pain*’ or ‘low* backpain*’ or ‘low* back ache*’ or ‘low* backache*’ or ‘low* back syndrome*’ or lumbago* or ‘lumbal pain*’ or ‘lumbal syndrome*’ or lumbalgia* or ‘lumbar pain*’ or ‘lumbar spine syndrome*’ or lumbodynia* or ‘lumbosacral pain*’ or ‘lumbar multifidus pain*’ or ‘lumbar flexion rotation syndrome*’ or ‘lumbar extension rotation syndrome*’ or LBP or CLBP or NSLBP) and (chronic or continu* or constant or persistent or prolonged or longstanding) and (electromyogra* or EMG or sEMG or kinematic* or angle). The search was based on the title, keywords and abstract. Duplicates were identified and removed using Mendeley (https://www.mendeley.com)^48^. Cross-referencing was also undertaken by checking references cited by the articles included in the full-text inspection.

### Study selection and eligibility criteria

The records identified from the search strategy were reviewed according to the eligibility criteria reported in Table 3.

**Table 3:** Eligibility criteria of the identified studies.

Properties	Eligibility criteria
Type of article	Original article (i.e. commentaries, letters to the editors, case reports, reviews, theses, and conference papers were excluded)
Language	English or French
Population	Adult patients (18–65 year-old) suffering from non-specific chronic^†^ low-back pain (NSCLBP) without a history of back surgery or pregnancy
Intervention	No intervention (e.g. rehabilitation, medication) on participants
Comparison	NSCLBP population compared with an asymptomatic control group
Outcomes	Movement and/or muscular activity related measurements

^†^A patient was defined chronic when pain duration was superior or equal to 3 months.

One author (FM) inspected the literature by screening records title and abstract using the Rayyan online application (http://rayyan.qcri.org), a tool developed to support the systematic review process^49^. Full-text inspection of the identified records was then undertaken independently by two authors (FM and KRD) to determine studies included in the analysis.

### Risk of bias assessment within studies

The risk of bias assessment within included studies was assessed using the modified McMaster Critical Review Form for Quantitative Studies^43^, already used in the context of low back pain^44^. This tool is based on 16 criteria: *C1: Purpose, C2: Literature review, C3: Study design, C4: Blinding, C5: Sample description, C6: Sample size, C7: Ethics and consent, C8: Validity of outcome, C9: Reliability of outcome, C10: Intervention description, C11: Statistical significance, C12: Statistical analysis, C13: Clinical importance, C14: Conclusions, C15: Clinical implications, C16: Study limitations.* Each criterion was rated one (satisfying description or justification) or zero (limited information or no information). In the current review, a score of ≥ 9 was used for an indication of acceptable methodological quality. Each included study was assessed independently by two authors (FM and KRD). In case of discrepancy, the original article was checked by a third author (SA).

### Data extraction

In the first stage of data extraction, the characteristics of the populations and measured parameters were extracted for each study. Populations (control and NSCLBP) were described by number of subjects, age, BMI, gender ratio, primary country of origin, as well as pain level (Visual Analog Scale^50^ results) and disability score (Oswestry Disability Index score^51^ or Roland Morris Disability Questionnaire^52^), when available. Parameters were defined by a type (movement or muscular activity), a variable and a task. Variables were described by a category (temporal, spatial/intensity, frequential, variability, coordination), a name, a unit, a measurement tool and a region of interest (e.g. multifidus, thorax). Tasks were described by an International Classification of Functioning, Disability and Health (ICF) 2nd level category^53^, a name, a movement constraint and a movement perturbation, when available. For each parameter, the measurement properties were extracted from the original article, when available, in term of three COSMIN domains^17^, namely reliability (intra-observer and inter-observer reliability), validity (content validity, criterion validity, construct validity) and interpretability (minimal detectable change), but also in term of clinical applicability (cost, simplicity of setup and execution, task-related pain)^54^. Content validity and clinical applicability were reported as poor/moderate/good/excellent levels depending on the number of weak items (reported between brackets). Specific test results were reported for reliability, other validity items and interpretability (e.g. interclass correlation, p-value). Two authors (FM and KRD) extracted data independently and cross-checked. In case of discrepancy, the original article was checked by a third author (SA).

In the second stage of data extraction, all parameters having demonstrated a construct validity at least moderate (see Table 4 for rating) were defined as biomarkers. When these biomarkers were measured in a sufficiently similar way in terms of variable and task, they were merged across studies to conduct a qualitative analysis for the data synthesis of these instruments.

**Table 4:** Rating used for each measurement property evaluated.

Rating	Reliability^56^	Content validity	Construct validity	Clinical applicability
Excellent	0.90 ≤ ICC	0 item^†^	*p* ≤ 0.001	0 item^‡^
Good	0.75 ≤ ICC < 0.90	1 item^†^	0.010 ≤ *p* < 0.001	1 item^‡^
Moderate	0.50 ≤ ICC < 0.75	2 items^†^	0.050 ≤ *p* < 0.010	2 items^‡^
Poor	ICC < 0.50	3 items^†^	0.05 < *p*	3 items^‡^

^†^Irrelevant or not sufficiently defined items regarding the construct to be assessed (e.g. population, instrument, parameter, outcome).

^‡^Weak items (e.g. cost, simplicity of setup and execution, task-related pain).

Each biomarker was identified by a code and, as for parameters, defined by a type, a variable and a task. The total number of subjects and patients, as well as the related studies, were also reported. To come to a clear, objective and informative overview of the identified biomarkers, the rating of their reliability, validity, interpretability and clinical applicability were then ranged from excellent, good, moderate to poor. The overall score for each measurement property of each biomarker was obtained by reporting the overall majority of the results obtained across related studies. The rating of each measurement property was defined in Table 4. Only the availability of minimum detectable change (MDC) was stated.

### Data synthesis

The targeted populations of the included studies were compiled on a labelled world map (generated with Microsoft Excel 2019, Microsoft Corporation, USA) to highlight in which countries NSCLBP was analysed, by how many studies and on how many participants (control and NSCLBP).

The assessment strategies observed in the included studies were highlighted in terms of parameter type, ICF 2nd level category, variable category and region of interest. For that, a Circos plot^55^ was generated to establish the links between each included study and these strategies.

The measurement properties of the identified biomarkers were highlighted using Circos plots^55^. The primary characteristics, i.e. corresponding ICF 2nd level category, variable category and region of interest, were also reported on these plots, while the full characteristics of each biomarker are available in Supplementary Table 3.

## Supplementary Information

Supplementary Information 1.

Supplementary Information 2.

Supplementary Information 3.

## References

[1] Vos T (2017). Global, regional, and national incidence, prevalence, and years lived with disability for 328 diseases and injuries for 195 countries, 1990–2016: a systematic analysis for the Global Burden of Disease Study 2016. The Lancet.

[2] Airaksinen O (2006). Chapter 4. European guidelines for the management of chronic nonspecific low back pain. Eur Spine J.

[3] Hartvigsen J (2018). What low back pain is and why we need to pay attention. The Lancet.

[4] Balagué F, Mannion AF, Pellisé F, Cedraschi C (2012). Non-specific low back pain. The Lancet.

[5] Foster NE (2018). Prevention and treatment of low back pain: evidence, challenges, and promising directions. The Lancet.

[6] Dubois JD, Abboud J, St-Pierre C, Piche M, Descarreaux M (2014). Neuromuscular adaptations predict functional disability independently of clinical pain and psychological factors in patients with chronic non-specific low back pain. J Electromyogr. Kinesiol..

[7] Ramond A (2011). Psychosocial risk factors for chronic low back pain in primary care–a systematic review. Fam. Pract..

[8] Ranger TA (2020). Catastrophization, fear of movement, anxiety, and depression are associated with persistent, severe low back pain and disability. Spine J..

[9] Hodges PW, Tucker K (2011). Moving differently in pain: a new theory to explain the adaptation to pain. Pain.

[10] Salvioli S, Pozzi A, Testa M (2019). Movement control impairment and low back pain: state of the art of diagnostic framing. Medicina (Kaunas).

[11] Koch C, Hansel F (2018). Chronic non-specific low back pain and motor control during gait. Front. Psychol..

[12] Cholewicki J (2019). Can biomechanics research lead to more effective treatment of low back pain? A point-counterpoint debate. J. Orthop. Sports Phys. Ther..

[13] Sadler S, Cassidy S, Peterson B, Spink M, Chuter V (2019). Gluteus medius muscle function in people with and without low back pain: a systematic review. BMC Musculoskelet. Disord..

[14] Hodges PW, Danneels L (2019). Changes in structure and function of the back muscles in low back pain: different time points, observations, and mechanisms. J. Orthop. Sports Phys. Ther..

[15] Rose-Dulcina K, Genevay S, Dominguez D, Armand S, Vuillerme N (2020). Flexion-relaxation ratio asymmetry and its relation with trunk lateral rom in individuals with and without chronic nonspecific low back pain. Spine Phila Pa (1976).

[16] Strimbu K, Tavel JA (2010). What are biomarkers?. Curr. Opin. HIV AIDS.

[17] Mokkink LB (2010). The COSMIN study reached international consensus on taxonomy, terminology, and definitions of measurement properties for health-related patient-reported outcomes. J. Clin. Epidemiol..

[18] Mehravar M (2012). Principal component analysis of kinematic patterns variability during sit to stand in people with non-specific chronic low back pain. J. Mech. Med. Biol..

[19] Muller R, Ertelt T, Blickhan R (2015). Low back pain affects trunk as well as lower limb movements during walking and running. J. Biomech..

[20] Vogt L, Pfeifer K, Banzer W (2003). Neuromuscular control of walking with chronic low-back pain. Man Ther..

[21] Kuriyama N, Ito H (2005). Electromyographic functional analysis of the lumbar spinal muscles with low back pain. J. Nippon Med. Sch..

[22] Coghlin SS, McFadyen BJ (1994). Transfer strategies used to rise from a chair in normal and low back pain subjects. Clin. Biomech..

[23] van Dijk MJH (2020). Assessment instruments of movement quality in patients with non-specific low back pain: a systematic review and selection of instruments. Gait Posture.

[24] Reeves NP, Cholewicki J, van Dieen JH, Kawchuk G, Hodges PW (2019). Are stability and instability relevant concepts for back pain?. J. Orthop. Sports Phys. Ther..

[25] Hidalgo B, Gilliaux M, Poncin W, Detrembleur C (2012). Reliability and validity of a kinematic spine model during active trunk movement in healthy subjects and patients with chronic non-specific low back pain. J. Rehabil. Med..

[26] Leardini A, Biagi F, Merlo A, Belvedere C, Benedetti MG (2011). Multi-segment trunk kinematics during locomotion and elementary exercises. Clin. Biomech. (Bristol, Avon).

[27] Shafizadeh M (2016). Movement coordination during sit-to-stand in low back pain people. Hum. Mov..

[28] Silfies SP, Bhattacharya A, Biely S, Smith SS, Giszter S (2009). Trunk control during standing reach: a dynamical system analysis of movement strategies in patients with mechanical low back pain. Gait Posture.

[29] Shum GLK, Crosbie J, Lee RYW (2005). Effect of low back pain on the kinematics and joint coordination of the lumbar spine and hip during sit-to-stand and stand-to-sit. Spine.

[30] Travell J, Rinzler S, Herman M (1942). Pain and disability of the shoulder and arm: treatment by intramuscular infiltration with procaine hydrochloride. J. Am. Med. Assoc..

[31] van Dieën JH, Selen LPJ, Cholewicki J (2003). Trunk muscle activation in low-back pain patients, an analysis of the literature. J. Electromyogr. Kinesiol..

[32] Mehta R, Cannella M, Smith SS, Silfies SP (2010). Altered trunk motor planning in patients with nonspecific low back pain. J. Mot. Behav..

[33] Liebetrau A, Puta C, Anders C, de Lussanet MH, Wagner H (2013). Influence of delayed muscle reflexes on spinal stability: model-based predictions allow alternative interpretations of experimental data. Hum. Mov. Sci..

[34] Abboud J (2014). Trunk motor variability in patients with non-specific chronic low back pain. Eur. J. Appl. Physiol..

[35] Mehta R (2017). Trunk postural muscle timing is not compromised in low back pain patients clinically diagnosed with movement coordination impairments. Mot. Control.

[36] Solnik S, DeVita P, Rider P, Long B, Hortobágyi T (2008). Teager–Kaiser operator improves the accuracy of EMG onset detection independent of signal-to-noise ratio. Acta Bioeng. Biomech..

[37] Neblett R, Brede E, Mayer TG, Gatchel RJ (2013). What is the best surface EMG measure of lumbar flexion-relaxation for distinguishing chronic low back pain patients from pain-free controls?. Clin. J. Pain.

[38] Watson PJ, Booker CK, Main CJ, Chen ACN (1997). Surface electromyography in the identification of chronic low back pain patients: the development of the flexion relaxation ratio. Clin. Biomech..

[39] Nougarou F, Massicotte D, Descarreaux M (2012). Detection method of flexion relaxation phenomenon based on wavelets for patients with low back pain. EURASIP J. Adv. Signal Process..

[40] Sarti MA, Lisón JF, Monfort M, Fuster MA (2001). Response of the flexion-relaxation phenomenon relative to the lumbar motion to load and speed. Spine.

[41] Pacher L, Chatellier C, Vauzelle R, Fradet L (2020). Sensor-to-segment calibration methodologies for lower-body kinematic analysis with inertial sensors: a systematic review. Sensors (Basel).

[42] Laird RA, Kent P, Keating JL (2016). How consistent are lordosis, range of movement and lumbo-pelvic rhythm in people with and without back pain?. BMC Musculoskelet. Disord..

[43] Takasaki H, Miki T (2017). The impact of continuous use of lumbosacral orthoses on trunk motor performance: a systematic review with meta-analysis. Spine J..

[44] Hori M, Hasegawa H, Takasaki H (2019). Comparisons of hamstring flexibility between individuals with and without low back pain: systematic review with meta-analysis. Physiother. Theory Pract..

[45] Hodges PW, van Dieen JH, Cholewicki J (2019). Time to reflect on the role of motor control in low back pain. J. Orthop. Sports Phys. Ther..

[46] Miller SA, Forrest JL (2001). Enhancing your practice through evidence-based decision making: PICO, learning how to ask good questions. J. Evid.-Based Dental Pract..

[47] Moher, D., Liberati, A., Tetzlaff, J., Altman, D. G. & Group, P (2009). Preferred reporting items for systematic reviews and meta-analyses: the PRISMA statement. PLoS Med..

[48] Elston DM (2019). Mendeley. J. Am. Acad. Dermatol..

[49] Ouzzani M, Hammady H, Fedorowicz Z, Elmagarmid A (2016). Rayyan-a web and mobile app for systematic reviews. Syst. Rev..

[50] Hawker GA, Mian S, Kendzerska T, French M (2011). Measures of adult pain: visual analog scale for pain (VAS pain), numeric rating scale for pain (NRS pain), McGill pain questionnaire (MPQ), short-form mcgill pain questionnaire (SF-MPQ), chronic pain grade scale (CPGS), short form-36 bodily pain scale (SF-36 BPS), and measure of intermittent and constant osteoarthritis pain (ICOAP). Arthritis Care Res. (Hoboken).

[51] Fairbank JCT, Pynsent PB (2000). The oswestry disability index. Spine.

[52] Roland M, Morris R (1983). A study of the natural history of back pain: part idevelopment of a reliable and sensitive measure of disability in low-back pain. Spine.

[53] World Health, O. (World Health Organization, Geneva, 2001).

[54] Villafane JH (2016). Validity and everyday clinical applicability of lumbar muscle fatigue assessment methods in patients with chronic non-specific low back pain: a systematic review. Disabil. Rehabil..

[55] Krzywinski M (2009). Circos: an information aesthetic for comparative genomics. Genome Res..

[56] Koo TK, Li MY (2016). A guideline of selecting and reporting intraclass correlation coefficients for reliability research. J. Chiropract. Med..

[57] Pourahmadi, M. R. *et al*. Test-retest reliability of sit-to-stand and stand-to-sit analysis in people with and without chronic non-specific low back pain. *Musculoskelet. Sci. Pract.* **35**, 95–104. 10.1016/j.msksp.2017.11.001 (2018). 10.1016/j.msksp.2017.11.001 29128293

[58] Hidalgo, B., Gobert, F., Bragard, D. & Detrembleur, C. Effects of proprioceptive disruption on lumbar spine repositioning error in a trunk forward bending task. *J Back Musculoskelet Rehabil* **26**, 381–387. 10.3233/BMR-130396 (2013). 10.3233/BMR-130396 23948825

[59] Haj, A., Weisman, A. & Masharawi, Y. Lumbar axial rotation kinematics in men with non-specific chronic low back pain.* Clin Biomech* **61**, 192–198. 10.1016/j.clinbiomech.2018.12.022 (2019). 10.1016/j.clinbiomech.2018.12.022 30594767

[60] Mokhtarinia, H. R., Sanjari, M. A., Chehrehrazi, M., Kahrizi, S. & Parnianpour, M. Trunk coordination in healthy and chronic nonspecific low back pain subjects during repetitive flexion-extension tasks: Effects of movement asymmetry, velocity and load. *Hum. Mov.* **45**, 182–192. 10.1016/j.humov.2015.11.007 (2016). 10.1016/j.humov.2015.11.007 26684726

[61] Ahern, D. K., Follick, M. J., Council, J. R., Laser-Wolston, N. & Litchman, H. Comparison of lumbar paravertebral EMG patterns in chronic low back pain patients and non-patient controls. *Pain ***34**, 153–160. 10.1016/0304-3959(88)90160-1 (1988). 10.1016/0304-3959(88)90160-1 2971912

[62] Larivière, C. *et al.* Specificity of a back muscle roman chair exercise in healthy and back pain subjects. *Med. Sci. Sports Exerc.* **43**, 157–164. 10.1249/MSS.0b013e3181e96388 (2011). 10.1249/MSS.0b013e3181e96388 20508534

[63] Matheve, T., De Baets, L., Bogaerts, K. & Timmermans, A. Lumbar range of motion in chronic low back pain is predicted by task-specific, but not by general measures of pain-related fear. * Eur J Pain (United Kingdom)*. 10.1002/ejp.1384 (2019). 10.1002/ejp.1384 30793429

[64] Jacobs, J. V., Roy, C. L., Hitt, J. R., Popov, R. E. & Henry, S. M. Neural mechanisms and functional correlates of altered postural responses to perturbed standing balance with chronic low back pain. *Neuroscience ***339**, 511–524. 10.1016/j.neuroscience.2016.10.032 (2016). 10.1016/j.neuroscience.2016.10.032 PMC5118100 27771534

[65] Osuka, S. *et al*. The onset of deep abdominal muscles activity during tasks with different trunk rotational torques in subjects with non-specific chronic low back pain. *J. Orthop. Sci*. 10.1016/j.jos.2018.12.028 (2019). 10.1016/j.jos.2018.12.028 30711377

[66] Ramprasad, M., Shenoy, D. S., Singh, S. J., Sankara, N. & Joseley, S. R. P. The magnitude of pre-programmed reaction dysfunction in back pain patients: Experimental pilot electromyography study. *J Back Musculoskelet Rehabil* **23**, 77–86. 10.3233/BMR-2010-0254 (2010). 10.3233/BMR-2010-0254 20555120

