# Supplementary material for: A systematic review of movement and muscular activity biomarkers to discriminate non-specific chronic low back pain patients from an asymptomatic population
Source: Sci Rep. 2021 Mar 12;11:5850. doi: 10.1038/s41598-021-84034-x (PMC7955136; doi:10.1038/s41598-021-84034-x)
Supplement: Supplementary file 1 — Supplementary Information 1. [file 41598_2021_84034_MOESM1_ESM.docx]

**Title**

A systematic review of movement and muscular activity biomarkers to discriminate non-specific chronic low back pain patients from an asymptomatic population.

**Author names**

Florent Moissenet^1*^, Kevin Rose-Dulcina^1^, Stéphane Armand^1^ and Stéphane Genevay^2^.

**Affiliations**

^1^ Kinesiology Laboratory, University of Geneva and Geneva University Hospitals, Geneva, Switzerland.
^2^ Department of Rheumatology, Geneva University Hospitals, Geneva, Switzerland.

**S1 Table.** **Results from the quality assessment of included articles.** Each criterion was rated one (satisfying description or justification) or zero (limited information or no information). The overall score of each article was calculated by the sum of rated criteria and in percentages. A score of ≥9 was used for an indication of acceptable methodological quality (articles with an overall score ≥9 have been highlighted). The total score (%) of each criterion across articles is also reported in the last row of the table.

| **Study** | **Criterion** | | | | | | | | | | | | | | | | **Sum** | **%** |
| --- | --- | --- | --- | --- | --- | --- | --- | --- | --- | --- | --- | --- | --- | --- | --- | --- | --- | --- |
|  | **1** | **2** | **3** | **4** | **5** | **6** | **7** | **8** | **9** | **10** | **11** | **12** | **13** | **14** | **15** | **16** |  |  |
| Abboud et al. (2014) | 1 | 1 | 0 | 0 | 1 | 0 | 1 | 1 | 0 | 1 | 1 | 1 | 0 | 1 | 1 | 1 | 11 | 69 |
| Ahern et al. (1988) | 1 | 1 | 0 | 1 | 1 | 0 | 0 | 1 | 0 | 1 | 0 | 0 | 1 | 1 | 1 | 0 | 9 | 56 |
| Ambroz et al. (2000) | 1 | 1 | 0 | 0 | 1 | 0 | 1 | 1 | 0 | 1 | 1 | 1 | 0 | 1 | 1 | 1 | 11 | 69 |
| Ansari et al. (2018) | 1 | 1 | 0 | 0 | 1 | 1 | 1 | 1 | 0 | 1 | 1 | 1 | 1 | 1 | 1 | 1 | 13 | 81 |
| Arendt-Nielsen et al. (1995) | 1 | 1 | 1 | 0 | 1 | 0 | 1 | 1 | 0 | 1 | 1 | 1 | 1 | 1 | 0 | 1 | 12 | 75 |
| Asgari et al. (2015) | 1 | 1 | 0 | 0 | 1 | 0 | 1 | 1 | 0 | 1 | 1 | 1 | 1 | 1 | 1 | 1 | 12 | 75 |
| Asgari et al. (2017) | 1 | 1 | 0 | 0 | 1 | 0 | 1 | 1 | 0 | 1 | 1 | 1 | 0 | 1 | 1 | 1 | 11 | 69 |
| Beneck et al. (2013) | 1 | 1 | 0 | 0 | 1 | 0 | 1 | 1 | 0 | 1 | 1 | 1 | 0 | 1 | 1 | 0 | 10 | 63 |
| Bourigua et al. (2014) | 1 | 1 | 1 | 0 | 1 | 0 | 1 | 0 | 0 | 1 | 1 | 1 | 1 | 1 | 1 | 1 | 12 | 75 |
| Chehrehrazi et al. (2017) | 1 | 1 | 0 | 0 | 1 | 0 | 1 | 1 | 0 | 1 | 1 | 1 | 1 | 1 | 1 | 1 | 12 | 75 |
| Christe et al. (2016) | 1 | 1 | 1 | 0 | 1 | 0 | 1 | 1 | 0 | 1 | 1 | 1 | 0 | 1 | 1 | 1 | 12 | 75 |
| Christe et al. (2017) | 1 | 1 | 1 | 0 | 1 | 0 | 1 | 1 | 1 | 1 | 1 | 1 | 1 | 1 | 1 | 1 | 14 | 88 |
| Coghlin et al. (1994) | 1 | 1 | 0 | 0 | 1 | 0 | 1 | 1 | 0 | 1 | 0 | 0 | 0 | 1 | 0 | 0 | 7 | 44 |
| Cohen et al. (1986) | 1 | 1 | 0 | 0 | 1 | 0 | 1 | 1 | 0 | 1 | 1 | 1 | 1 | 1 | 1 | 0 | 11 | 69 |
| Courbalay et al. (2017) | 1 | 1 | 1 | 0 | 1 | 0 | 1 | 1 | 0 | 1 | 1 | 1 | 1 | 1 | 1 | 1 | 13 | 81 |
| Crossman et al. (2004) | 1 | 1 | 1 | 0 | 1 | 1 | 1 | 1 | 0 | 1 | 1 | 1 | 1 | 1 | 1 | 0 | 13 | 81 |
| da Silva et al. (2019) | 1 | 1 | 0 | 0 | 1 | 1 | 1 | 1 | 0 | 1 | 1 | 1 | 1 | 1 | 1 | 1 | 13 | 81 |
| Dankaerts et al. (2004) | 1 | 1 | 0 | 0 | 1 | 0 | 1 | 1 | 1 | 1 | 1 | 1 | 0 | 1 | 0 | 1 | 11 | 69 |
| Dankaerts et al. (2006a) | 1 | 1 | 1 | 0 | 1 | 0 | 1 | 1 | 0 | 1 | 1 | 1 | 1 | 1 | 1 | 1 | 13 | 81 |
| Dankaerts et al. (2006b) | 1 | 1 | 1 | 0 | 1 | 0 | 1 | 1 | 1 | 1 | 1 | 1 | 1 | 1 | 1 | 1 | 14 | 88 |
| Dankaerts et al. (2009) | 1 | 1 | 1 | 1 | 1 | 1 | 1 | 1 | 1 | 1 | 1 | 1 | 1 | 1 | 1 | 1 | 16 | 100 |
| Descarreaux et al. (2007) | 1 | 1 | 0 | 0 | 1 | 0 | 1 | 0 | 0 | 1 | 1 | 1 | 1 | 1 | 1 | 1 | 11 | 69 |
| Du et al. (2018) | 1 | 1 | 0 | 0 | 1 | 0 | 1 | 0 | 0 | 1 | 1 | 1 | 1 | 1 | 1 | 1 | 11 | 69 |
| Ebrahimi et al. (2017) | 1 | 1 | 1 | 0 | 1 | 0 | 1 | 1 | 0 | 1 | 1 | 1 | 0 | 1 | 1 | 0 | 11 | 69 |
| Falla et al. (2014) | 1 | 1 | 0 | 0 | 1 | 0 | 1 | 0 | 0 | 1 | 1 | 1 | 1 | 1 | 1 | 1 | 11 | 69 |
| Gombatto et al. (2008) | 1 | 1 | 0 | 0 | 1 | 0 | 1 | 1 | 1 | 1 | 1 | 1 | 1 | 1 | 1 | 1 | 13 | 81 |
| Götze et al. (2015) | 1 | 1 | 0 | 1 | 1 | 0 | 1 | 0 | 0 | 1 | 1 | 1 | 0 | 1 | 1 | 0 | 10 | 63 |
| Hadizadeh et al. (2014) | 1 | 1 | 1 | 0 | 1 | 0 | 1 | 0 | 0 | 1 | 1 | 1 | 1 | 1 | 1 | 1 | 12 | 75 |
| Haj et al. (2019) | 1 | 1 | 0 | 0 | 1 | 0 | 1 | 1 | 1 | 1 | 1 | 1 | 1 | 1 | 1 | 1 | 13 | 81 |
| Healey et al. (2005) | 1 | 1 | 1 | 0 | 1 | 0 | 1 | 0 | 0 | 1 | 1 | 1 | 0 | 1 | 1 | 0 | 10 | 63 |
| Hemmati et al. (2018) | 1 | 1 | 1 | 0 | 1 | 0 | 1 | 1 | 0 | 1 | 1 | 1 | 0 | 1 | 1 | 0 | 11 | 69 |
| Hemming et al. (2018) | 1 | 1 | 1 | 0 | 1 | 1 | 1 | 0 | 1 | 1 | 1 | 1 | 1 | 1 | 1 | 1 | 14 | 88 |
| Henry et al. (2006) | 1 | 1 | 0 | 0 | 1 | 0 | 1 | 0 | 0 | 1 | 1 | 1 | 1 | 0 | 1 | 1 | 10 | 63 |
| Hidalgo et al. (2012) | 1 | 1 | 1 | 0 | 1 | 0 | 1 | 1 | 1 | 1 | 1 | 1 | 1 | 1 | 1 | 1 | 14 | 88 |
| Hidalgo et al. (2013) | 1 | 1 | 0 | 0 | 1 | 0 | 1 | 1 | 1 | 1 | 1 | 1 | 0 | 1 | 1 | 1 | 12 | 75 |
| Himmelreich et al. (2008) | 1 | 1 | 0 | 0 | 1 | 0 | 1 | 1 | 0 | 1 | 1 | 1 | 0 | 1 | 1 | 0 | 10 | 63 |
| Hodges (2001) | 1 | 1 | 0 | 0 | 1 | 0 | 1 | 1 | 0 | 1 | 1 | 1 | 1 | 1 | 1 | 0 | 11 | 69 |
| Hu et al. (2010) | 1 | 1 | 0 | 0 | 1 | 0 | 1 | 1 | 0 | 1 | 1 | 1 | 1 | 1 | 1 | 0 | 11 | 69 |
| Hubley-Kozey and Vezina (2002) | 1 | 1 | 1 | 0 | 1 | 0 | 1 | 1 | 0 | 1 | 1 | 1 | 0 | 1 | 1 | 1 | 12 | 75 |
| Humphrey et al. (2005) | 1 | 1 | 0 | 1 | 1 | 0 | 0 | 1 | 0 | 1 | 1 | 1 | 0 | 1 | 1 | 0 | 10 | 63 |
| Jacobs et al. (2011) | 1 | 1 | 0 | 0 | 1 | 0 | 1 | 1 | 0 | 1 | 1 | 1 | 1 | 1 | 0 | 1 | 11 | 69 |
| Jacobs et al. (2016) | 1 | 1 | 0 | 0 | 1 | 1 | 1 | 1 | 0 | 1 | 1 | 1 | 1 | 1 | 0 | 0 | 11 | 69 |
| Kankaanpää et al. (1998) | 1 | 1 | 1 | 0 | 1 | 0 | 1 | 1 | 0 | 1 | 1 | 1 | 1 | 1 | 1 | 0 | 12 | 75 |
| Kankaanpää et al. (2005) | 1 | 1 | 0 | 0 | 1 | 0 | 1 | 0 | 0 | 1 | 1 | 1 | 0 | 1 | 0 | 1 | 9 | 56 |
| Kim et al. (2014a) | 1 | 1 | 0 | 0 | 1 | 0 | 1 | 1 | 0 | 1 | 1 | 0 | 1 | 1 | 0 | 1 | 10 | 63 |
| Kim et al. (2014b) | 1 | 1 | 1 | 0 | 1 | 0 | 1 | 1 | 0 | 1 | 1 | 0 | 1 | 1 | 0 | 1 | 11 | 69 |
| Kuriyama and Ito (2005) | 1 | 0 | 0 | 0 | 1 | 0 | 0 | 1 | 0 | 0 | 0 | 0 | 0 | 1 | 0 | 0 | 4 | 25 |
| Laird et al. (2016) | 1 | 1 | 0 | 0 | 1 | 1 | 1 | 1 | 1 | 1 | 1 | 1 | 0 | 1 | 1 | 1 | 13 | 81 |
| Lamoth et al. (2006a) | 1 | 1 | 1 | 0 | 1 | 0 | 1 | 1 | 0 | 1 | 1 | 1 | 1 | 1 | 1 | 0 | 11 | 69 |
| Lamoth et al. (2006b) | 1 | 1 | 0 | 0 | 1 | 0 | 1 | 1 | 0 | 1 | 1 | 1 | 1 | 1 | 1 | 1 | 12 | 75 |
| Larivière et al. (2002) | 1 | 1 | 1 | 0 | 1 | 0 | 1 | 1 | 1 | 1 | 1 | 1 | 0 | 1 | 1 | 1 | 13 | 81 |
| Larivière et al. (2010) | 1 | 1 | 1 | 0 | 1 | 0 | 1 | 1 | 1 | 1 | 1 | 1 | 1 | 1 | 1 | 1 | 14 | 88 |
| Larivière et al. (2011) | 1 | 1 | 0 | 0 | 1 | 1 | 1 | 1 | 1 | 1 | 1 | 1 | 1 | 1 | 1 | 1 | 14 | 88 |
| Leinonen et al. (2000) | 1 | 1 | 1 | 0 | 1 | 0 | 1 | 1 | 0 | 1 | 1 | 1 | 1 | 0 | 1 | 0 | 11 | 69 |
| Leinonen et al. (2007) | 1 | 1 | 1 | 0 | 1 | 0 | 1 | 1 | 0 | 1 | 1 | 1 | 0 | 1 | 1 | 1 | 12 | 75 |
| Liebetrau et al. (2013) | 1 | 1 | 0 | 0 | 1 | 0 | 1 | 0 | 0 | 1 | 1 | 1 | 1 | 1 | 1 | 1 | 11 | 69 |
| Lima et al. (2018) | 1 | 1 | 0 | 1 | 1 | 1 | 1 | 1 | 1 | 1 | 1 | 1 | 0 | 1 | 0 | 1 | 13 | 81 |
| Lui et al. (2018) | 1 | 1 | 1 | 0 | 1 | 0 | 1 | 1 | 1 | 1 | 1 | 1 | 1 | 1 | 1 | 1 | 14 | 88 |
| Matheve et al. (2019) | 1 | 1 | 1 | 0 | 1 | 0 | 1 | 1 | 1 | 1 | 1 | 1 | 1 | 1 | 0 | 1 | 13 | 81 |
| McCaskey et al. (2018) | 1 | 1 | 1 | 0 | 1 | 0 | 1 | 0 | 0 | 1 | 1 | 1 | 0 | 1 | 1 | 1 | 11 | 69 |
| McDowell et al. (2018) | 1 | 1 | 0 | 0 | 1 | 1 | 1 | 1 | 1 | 1 | 1 | 1 | 1 | 1 | 1 | 1 | 14 | 88 |
| Mehravar et al. (2012) | 1 | 1 | 0 | 0 | 1 | 0 | 1 | 0 | 0 | 1 | 0 | 0 | 0 | 1 | 0 | 0 | 6 | 38 |
| Mehta et al. (2000) | 1 | 1 | 0 | 0 | 1 | 0 | 1 | 1 | 0 | 1 | 1 | 1 | 1 | 1 | 1 | 1 | 12 | 75 |
| Mokhtarinia et al. (2016) | 1 | 1 | 0 | 0 | 1 | 0 | 1 | 1 | 0 | 1 | 1 | 1 | 0 | 1 | 0 | 1 | 10 | 63 |
| Muller et al. (2015) | 1 | 1 | 0 | 0 | 0 | 0 | 1 | 0 | 0 | 1 | 0 | 0 | 0 | 0 | 1 | 1 | 6 | 38 |
| Neblett et al.(2014) | 1 | 1 | 0 | 0 | 1 | 0 | 1 | 1 | 0 | 1 | 0 | 0 | 1 | 1 | 1 | 1 | 10 | 63 |
| Ohe et al. (2015) | 1 | 1 | 1 | 0 | 1 | 0 | 1 | 1 | 1 | 1 | 1 | 1 | 1 | 1 | 1 | 1 | 14 | 88 |
| Osuka et al. (2019) | 1 | 1 | 0 | 0 | 1 | 1 | 1 | 1 | 0 | 1 | 1 | 1 | 0 | 1 | 0 | 1 | 11 | 69 |
| Pääsuke et al. (2002) | 1 | 1 | 0 | 1 | 1 | 0 | 1 | 1 | 0 | 1 | 1 | 1 | 0 | 1 | 1 | 0 | 11 | 69 |
| Pakzad et al. (2016) | 1 | 0 | 1 | 0 | 1 | 0 | 1 | 1 | 0 | 1 | 1 | 1 | 0 | 1 | 0 | 1 | 10 | 63 |
| Parfrey et al. (2014) | 1 | 1 | 0 | 0 | 1 | 0 | 1 | 1 | 1 | 1 | 1 | 1 | 1 | 1 | 1 | 1 | 14 | 88 |
| Penney et al. (2014) | 1 | 1 | 1 | 0 | 1 | 1 | 1 | 1 | 0 | 1 | 0 | 0 | 1 | 1 | 1 | 1 | 12 | 75 |
| Pirouzi et al. (2006) | 1 | 1 | 1 | 0 | 1 | 0 | 1 | 0 | 1 | 1 | 1 | 1 | 1 | 1 | 1 | 1 | 13 | 81 |
| Pourahmadi et al. (2017) | 1 | 1 | 1 | 0 | 0 | 1 | 1 | 0 | 1 | 1 | 1 | 1 | 0 | 1 | 1 | 1 | 12 | 75 |
| Pranata et al. (2019) | 1 | 1 | 1 | 0 | 1 | 0 | 1 | 1 | 0 | 1 | 1 | 1 | 0 | 1 | 0 | 1 | 11 | 69 |
| Radebold (2000) | 1 | 1 | 1 | 0 | 1 | 1 | 1 | 0 | 1 | 1 | 1 | 0 | 0 | 1 | 1 | 0 | 11 | 69 |
| Radebold (2001) | 1 | 1 | 1 | 0 | 1 | 0 | 1 | 1 | 1 | 1 | 1 | 1 | 0 | 1 | 1 | 0 | 12 | 75 |
| Ramprasad et al. (2010) | 1 | 1 | 1 | 0 | 1 | 0 | 1 | 1 | 1 | 1 | 1 | 1 | 0 | 1 | 1 | 1 | 13 | 81 |
| Ringheim et al. (2019) | 1 | 1 | 0 | 0 | 1 | 0 | 1 | 1 | 0 | 1 | 1 | 1 | 0 | 1 | 1 | 1 | 11 | 69 |
| Rossi et al. (2017) | 1 | 1 | 1 | 0 | 1 | 1 | 1 | 1 | 0 | 0 | 1 | 0 | 1 | 1 | 1 | 1 | 12 | 75 |
| Sheeran et al. (2012) | 1 | 1 | 1 | 0 | 1 | 1 | 1 | 1 | 1 | 1 | 1 | 1 | 1 | 1 | 1 | 1 | 15 | 94 |
| Suheiro et al. (2015) | 1 | 1 | 0 | 0 | 0 | 1 | 1 | 1 | 1 | 1 | 1 | 1 | 0 | 1 | 1 | 1 | 12 | 75 |
| Sung et al. (2013) | 1 | 1 | 0 | 0 | 1 | 0 | 1 | 1 | 0 | 1 | 1 | 1 | 1 | 1 | 1 | 1 | 12 | 75 |
| Suter et al. (2001) | 1 | 1 | 1 | 0 | 1 | 0 | 1 | 1 | 0 | 1 | 1 | 0 | 0 | 1 | 1 | 0 | 10 | 63 |
| Süüden et al. (2008) | 1 | 1 | 0 | 0 | 1 | 0 | 1 | 1 | 0 | 1 | 1 | 1 | 0 | 1 | 1 | 1 | 11 | 69 |
| Taghizadeh et al. (2017) | 1 | 1 | 1 | 1 | 1 | 1 | 1 | 1 | 0 | 1 | 1 | 1 | 1 | 1 | 1 | 1 | 15 | 94 |
| Tajali et al. (2013) | 1 | 1 | 0 | 0 | 1 | 0 | 1 | 1 | 0 | 1 | 1 | 1 | 0 | 1 | 0 | 1 | 10 | 63 |
| Taniguchi et al. (2017) | 1 | 1 | 1 | 0 | 1 | 0 | 1 | 1 | 0 | 1 | 1 | 1 | 0 | 1 | 0 | 1 | 11 | 69 |
| Vaisy et al. (2015) | 0 | 1 | 0 | 0 | 1 | 0 | 1 | 1 | 0 | 1 | 1 | 1 | 1 | 1 | 1 | 0 | 10 | 63 |
| Van der Huslt et al. (2010a) | 1 | 1 | 1 | 1 | 1 | 0 | 1 | 1 | 0 | 1 | 1 | 1 | 1 | 1 | 1 | 1 | 14 | 88 |
| Van der Huslt et al. (2010b) | 1 | 1 | 1 | 0 | 1 | 1 | 1 | 1 | 0 | 1 | 1 | 1 | 0 | 1 | 0 | 1 | 12 | 75 |
| Van Dieën et al. (2003) | 1 | 1 | 1 | 0 | 1 | 0 | 1 | 1 | 0 | 1 | 1 | 1 | 0 | 1 | 1 | 0 | 11 | 69 |
| Vogt et al. (2001) | 1 | 1 | 1 | 0 | 1 | 0 | 0 | 0 | 0 | 1 | 1 | 1 | 0 | 1 | 1 | 0 | 9 | 56 |
| Vogt et al. (2003) | 1 | 1 | 0 | 0 | 1 | 0 | 0 | 1 | 0 | 1 | 0 | 0 | 0 | 1 | 1 | 0 | 7 | 44 |
| Watson et al. (1997) | 1 | 1 | 0 | 0 | 1 | 0 | 1 | 1 | 1 | 1 | 1 | 0 | 1 | 1 | 1 | 0 | 11 | 69 |
| Wei et al. (2019) | 1 | 1 | 1 | 1 | 1 | 0 | 1 | 1 | 0 | 1 | 1 | 1 | 0 | 1 | 1 | 1 | 13 | 81 |
| Yoon et al. (2015) | 1 | 1 | 0 | 0 | 1 | 1 | 1 | 1 | 0 | 1 | 1 | 1 | 0 | 1 | 1 | 1 | 12 | 75 |
| Zhang et al. (2018) | 1 | 1 | 0 | 0 | 1 | 0 | 1 | 0 | 0 | 1 | 1 | 1 | 0 | 1 | 1 | 1 | 10 | 63 |
| **TOTAL (%)** | 99 | 98 | 46 | 9 | 97 | 20 | 95 | 81 | 28 | 98 | 92 | 86 | 53 | 97 | 81 | 71 | - | - |

***C1****: Purpose,* ***C2****: Literature review,* ***C3****: Study design,* ***C4****: Blinding,* ***C5****: Sample description,* ***C6****: Sample size,* ***C7****: Ethics and consent,* ***C8****: Validity of outcome,* ***C9****: Reliability of outcome,* ***C10****: Intervention description,* ***C11****: Statistical significance,* ***C12****: Statistical analysis,* ***C13****: Clinical importance,* ***C14****: Conclusions,* ***C15****: Clinical implications,* ***C16****: Study limitations*
